# Off-Target Integron Activity Leads to Rapid Plasmid Compensatory Evolution in Response to Antibiotic Selection Pressure

**DOI:** 10.1128/mbio.02537-22

**Published:** 2023-02-22

**Authors:** Célia Souque, José A. Escudero, R. Craig MacLean

**Affiliations:** a University of Oxford, Department of Biology, Oxford, United Kingdom; b Harvard Medical School, Department of Biomedical Informatics, Boston, Massachusetts, USA; c Harvard Medical School, Laboratory of Systems Pharmacology, Boston, Massachusetts, USA; d Universidad Complutense de Madrid, Departamento de Sanidad Animal, Madrid, Spain; e Universidad Complutense de Madrid, VISAVET, Madrid, Spain; LMU Munich

**Keywords:** antibiotic resistance, drug resistance evolution, integrons, mobile genetic elements, plasmid-mediated resistance

## Abstract

Integrons are mobile genetic elements that have played an important role in the dissemination of antibiotic resistance. Under stress, the integron can generate combinatorial variation in resistance cassette expression by cassette reshuffling, accelerating the evolution of resistance. However, the flexibility of the integron integrase site recognition motif hints at potential off-target effects of the integrase on the rest of the genome that may have important evolutionary consequences. Here, we test this hypothesis by selecting for increased-piperacillin-resistance populations of Pseudomonas aeruginosa with a mobile integron containing a difficult-to-mobilize β-lactamase cassette to minimize the potential for adaptive cassette reshuffling. We found that integron activity can decrease the overall survival rate but also improve the fitness of the surviving populations. Off-target inversions mediated by the integron accelerated plasmid adaptation by disrupting costly conjugative genes otherwise mutated in control populations lacking a functional integrase. Plasmids containing integron-mediated inversions were associated with lower plasmid costs and higher stability than plasmids carrying mutations albeit at the cost of a reduced conjugative ability. These findings highlight the potential for integrons to create structural variation that can drive bacterial evolution, and they provide an interesting example showing how antibiotic pressure can drive the loss of conjugative genes.

## INTRODUCTION

Mobile integrons are genetic shuffling devices heavily involved in the spread of antibiotic resistance (1). They consist of an integrase gene followed by an array of promoterless gene cassettes (2), predominantly encoding resistance genes (3). Cassettes are expressed from a promoter located at the end of the integrase gene such that their expression level is dependent on their position within the array (4, 5). When bacteria are exposed to antibiotics, the generation of DNA damage activates the SOS response and the expression of the integron integrase (6). The integrase enzyme then leads to cassette reshuffling, duplication, and deletion, generating combinatorial variation in both cassette presence/absence and expression levels, modulating bacterial antibiotic resistance levels (5, 7).

This cassette shuffling is possible through the recognition by the integrase of two motifs found within the integron: the double-stranded *attI* sites (8), located at the start of the array, and the single-stranded *attC* sites (9), located at the end of the cassettes. Recombination between two *attC* sites leads to the excision of the intervening cassette (10), while the *attC* × *attI* reaction promotes the integration of the cassette at the start of the array (11). Integrase recognition of these binding sites requires little sequence homology, relying instead on short degenerated core sequences (for the *attI* sites) (12) or the recognition of two to three extrahelical bases acting as structural landmarks (*attC* sites) (13). This flexibility creates the potential for the integron to generate “off-target” recombination with sites outside the integron, such as the insertion of the integron cassette into other parts of the genome (14, 15). These off-target effects of the integrase are likely to be deleterious: they may lead to the formation of chromosomal dimers that have to be resolved before cell division or potentially create genomic instability through the deletion of entire genomic regions (16). These reactions may therefore compromise the evolutionary benefits associated with integron cassette rearrangements. Moreover, these shuffling evolutionary benefits are likely to be dependent on the presence of highly mobile cassettes with strong positional effects on expression levels. As the mobility of integron cassettes is highly variable, with cassette recombination frequencies spanning several orders of magnitude depending on their *attC* site and their position within the array (17–19), the nature of the integron cassette cargo is likely to play a key role in shaping the relative importance of the on-target and off-target effects of the integron.

Our previous work investigating the evolutionary benefits of integrase activity used a model system involving a highly mobile *aadB* cassette that was under strong selection for an optimal position (5). Here, we test the evolutionary benefits of integron activity using *bla*_VEB-1_, a β-lactam resistance cassette shown to have low mobility (*attC_bla_*_VEB-1_ is 50 times less efficient for recombination than *attC_aadB_* [17]) and weak positional effects on antibiotic resistance. Under these conditions, the adaptive value of cassette reshuffling is likely to be low, and side effects of integrase activity are expected to play a greater role in determining the evolutionary impact of integron activity. In this scenario, we observed that integrase activity decreased overall population survival but had a beneficial impact on the fitness of the surviving populations through the generation of extensive plasmid backbone rearrangements, creating novel evolutionary pathways.

## RESULTS

### The impact of cassette position varies between cassettes.

**FIG 1:**
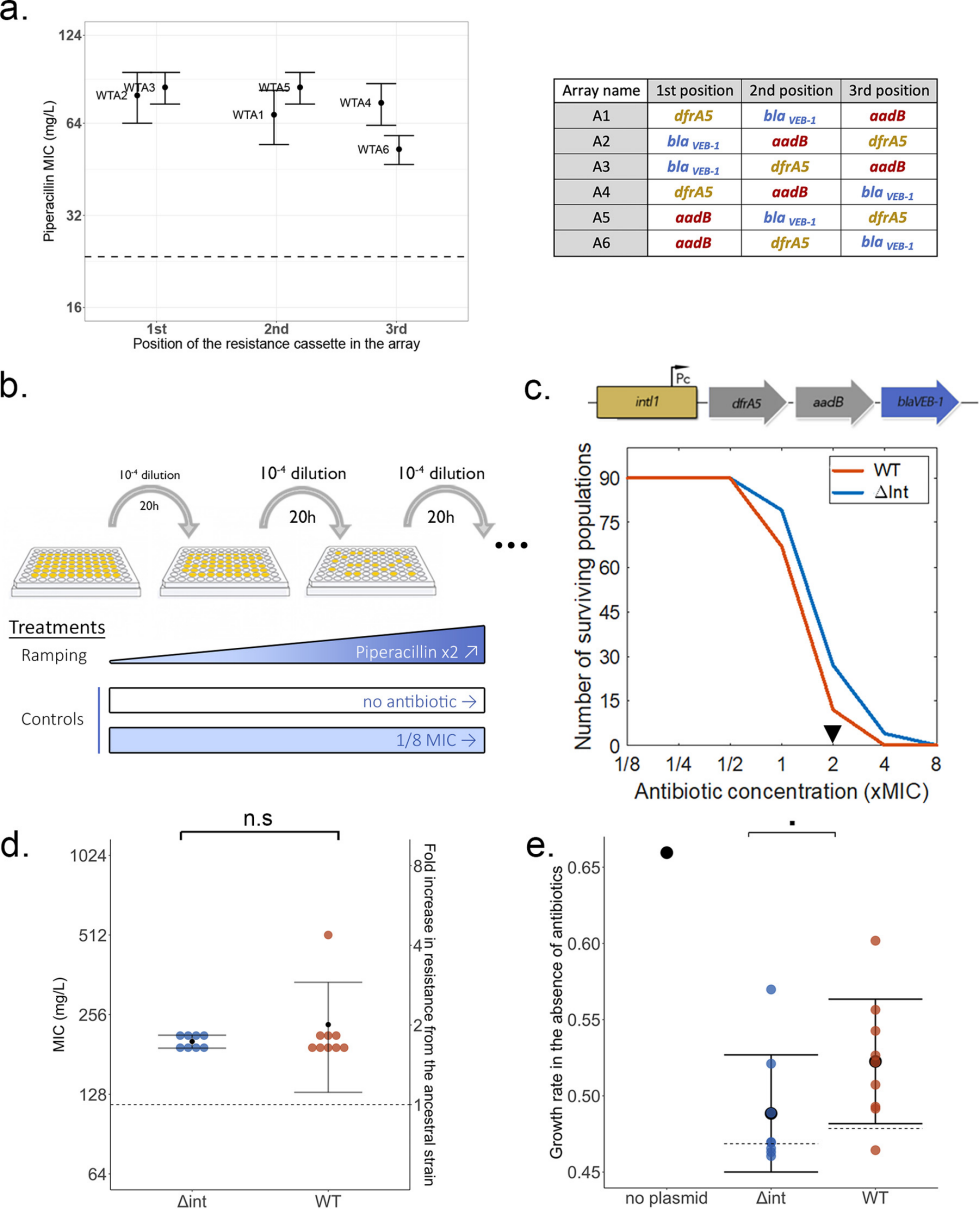
Integrase activity impacts bacterial evolution under antibiotic selection without altering resistance phenotypes. (a) Impact of the *bla*_VEB-1_ cassette position on resistance levels (piperacillin MIC) using a combinatorial integron array system (right). Error bars represent the standard errors from three biological replicates. The dotted line represents the P. aeruginosa resistance level in the absence of the plasmid. (b) Evolutionary ramp experimental design. (c, top) Representation of the WTA4 array expressed by the promoter Pc, with the β-lactamase cassette *bla*_VEB-1_ highlighted in blue. (Bottom) Survival rates of the WT and Δ*int* populations over time. The black triangle represents the time point selected for further phenotypic and genomic analysis. (d) Final piperacillin resistance levels of evolved populations sampled from the 2× MIC time point. Each dot represents the average from three independent biological replicates. The dotted line corresponds to the resistance level of the ancestral strains. Error bars represent standard deviations. Differences in resistance between the two genotypes were compared using a Fisher *t* test (*t* = −0.91247, df = 8.214, and *P* = 0.3875). n.s, not significant. (e) Final intrinsic growth rates in the absence of antibiotics of the evolved populations (per hour) as well as the ancestral population in the absence of the plasmid. Each dot represents the average from three independent biological replicates, with the dotted line corresponding to the growth rate of the ancestral strains. Error bars represent standard deviations. Differences in growth rates in the evolved populations between the two genotypes were compared using a Wilcoxon rank sum test (*W* = 16 and *P* = 0.059 [■]).

We used a combinatorial three-cassette class 1 integron system consisting of all six possible configurations of three resistance cassettes, *dfrA5*, *aadB*, and *bla*_VEB-1_, each encoding resistance to a different antibiotic family (trimethoprim, aminoglycosides, and β-lactams) (5), and measured the impact of cassette position on resistance levels. While previous results showed a strong impact with the *aadB* cassette of cassette position on resistance to gentamicin (5), the impact of position was much more subtle (Fig. 1a) or nonexistent (trimethoprim MIC of ≥1,500 mg/L for all arrays) when looking at piperacillin and trimethoprim resistance and the *bla*_VEB-1_ and *dfrA5* cassettes, respectively. At the transcription level, as expected, cassette position had an overall clear impact on transcript levels. However, and perhaps surprisingly, the impact of cassette position on transcription was variable depending on the cassettes and was weaker for *dfrA5* and *bla*_VEB-1_ than for the *aadB* cassette (see Fig. S1 in the supplemental material). This result could be explained by the presence of internal promoters within *dfrA5* and *bla*_VEB-1_, leading to the creation of partial transcripts. Adding to previous observations that the gentamicin resistance gradient provided by the *aadB* cassette was complemented by a decrease in translation, these results show that the expression of mobile integron cassettes and the resistance levels that they provide are the products of interactions among transcription, translation, and the nature of the resistance mechanism itself.

10.1128/mbio.02537-22.2 FIG S1 Transcript levels of each integron cassette depending on its position within the array relative to the cassette with the highest expression level. *aadB* expression data were reported previously (5) and are complemented with expression data for the two other cassettes. Cassette transcript levels are normalized based on two reference genes and the plasmid copy number. Error bars represent the standard errors from three independent biological replicates. Data for array WTA2 were excluded due to anomalously low levels of cassette transcription linked with disproportionately high integrase transcription levels, potentially due to an undetected mutation in the promoter region. Download FIG S1, TIF file, 3.9 MB. Copyright © 2023 Souque et al. 2023 Souque et al. https://creativecommons.org/licenses/by/4.0/ This content is distributed under the terms of the Creative Commons Attribution 4.0 International license.

The “adaptation-on-demand” model of the integron as an evolutionary catalyst assumes that antibiotic pressures impose strong selection for resistance cassettes to be positioned in the first position, as we have reported for *aadB*. However, these results show that cassette position can have weak effects on resistance levels provided by cassettes such as *bla*_VEB-1_ and *dfrA5*.

### Integrase activity impacts bacterial evolution under antibiotic selection without altering resistance phenotypes.

Given the various impacts of cassette position on resistance levels, we aimed to test the impact of the integron on resistance evolution when selection for cassette rearrangement is weak. Starting from array WTA4, which contains the *bla*_VEB-1_ cassette in the last position, we built a mutant with a dysfunctional integrase, Δ*intA4*, but providing similar initial levels of resistance. Using an “evolutionary ramp” design (20), we compared the abilities of both strains to evolve resistance to increasing concentrations of the antibiotic piperacillin (Fig. 1b). We passaged 90 independent populations of each strain for 7 days with doubling concentrations of antibiotics, starting from 1/8 times the MIC and ending at 8 times the original MIC. As a control, 15 populations of each strain were passaged either with low but constant selection (constant piperacillin concentration at 1/8 the MIC) or in the absence of selection (no antibiotic).

We monitored survival rates using optical density at 600 nm (OD_600_) readings and observed resistance evolution (survival at 1× MIC and higher) in most but not all populations, with the extinction of all populations at 8 times the original MIC (Fig. 1c). Surprisingly, we observed a higher survival rate of the Δ*int* populations, with some populations surviving at up to 4× MIC. While minor, the difference in survival rates was statistically significant (chi-square value = 12.4 on 1 degree of freedom and *P* = 4e−04 [by a log rank test]).

To test the impact of integrase activity on the evolution of resistance, we measured both piperacillin resistance and fitness in the absence of antibiotics for a subset of wild-type (WT) and Δ*int* evolved populations that evolved at up to 2× MIC (Fig. 1d and e). We observed no difference in final piperacillin resistance between the two genotypes (average MIC of WT populations of 235 ± 104 mg/L, versus 202 ± 11 mg/L for Δ*int* populations [*t* = −0.91247, df = 8.214, and *P* = 0.3875 {by a Fisher *t* test}]), suggesting that the integrase had no effect on the evolution of resistance *per se* (Fig. 1d). However, we were able to identify a small increase in final fitness in populations with a functional integrase (Fig. 1e) (average intrinsic growth rate of WT populations of 0.52 ± 0.04 h^−1^, versus 0.48 ± 0.038 h^−1^ for Δ*int* populations [*W* = 16 and *P* = 0.059 {by a Wilcoxon rank sum test}]), suggesting that integrase activity could open evolutionary pathways leading either to antibiotic resistance linked with a reduced fitness trade-off or overall improved fitness, for example, by alleviating the cost of plasmid carriage observed in the ancestral populations (Fig. 1e).

### Integron activity shapes plasmid evolution through extensive structural rearrangements.

We then investigated the genotype of the evolved ramping populations at 2× MIC using short-read (Illumina) whole-genome sequencing (Table S1). We observed a strong selection signal, with averages of 0.36 (WT) and 0.38 (Δ*int*) nonsynonymous mutations per population, for 0.033 (WT) and 0 (Δ*int*) synonymous mutations.

10.1128/mbio.02537-22.5 TABLE S1 List of mutations and rearrangements identified by sequencing. Download Table S1, XLSX file, 0.04 MB. Copyright © 2023 Souque et al. 2023 Souque et al. https://creativecommons.org/licenses/by/4.0/ This content is distributed under the terms of the Creative Commons Attribution 4.0 International license.

**FIG 2:**
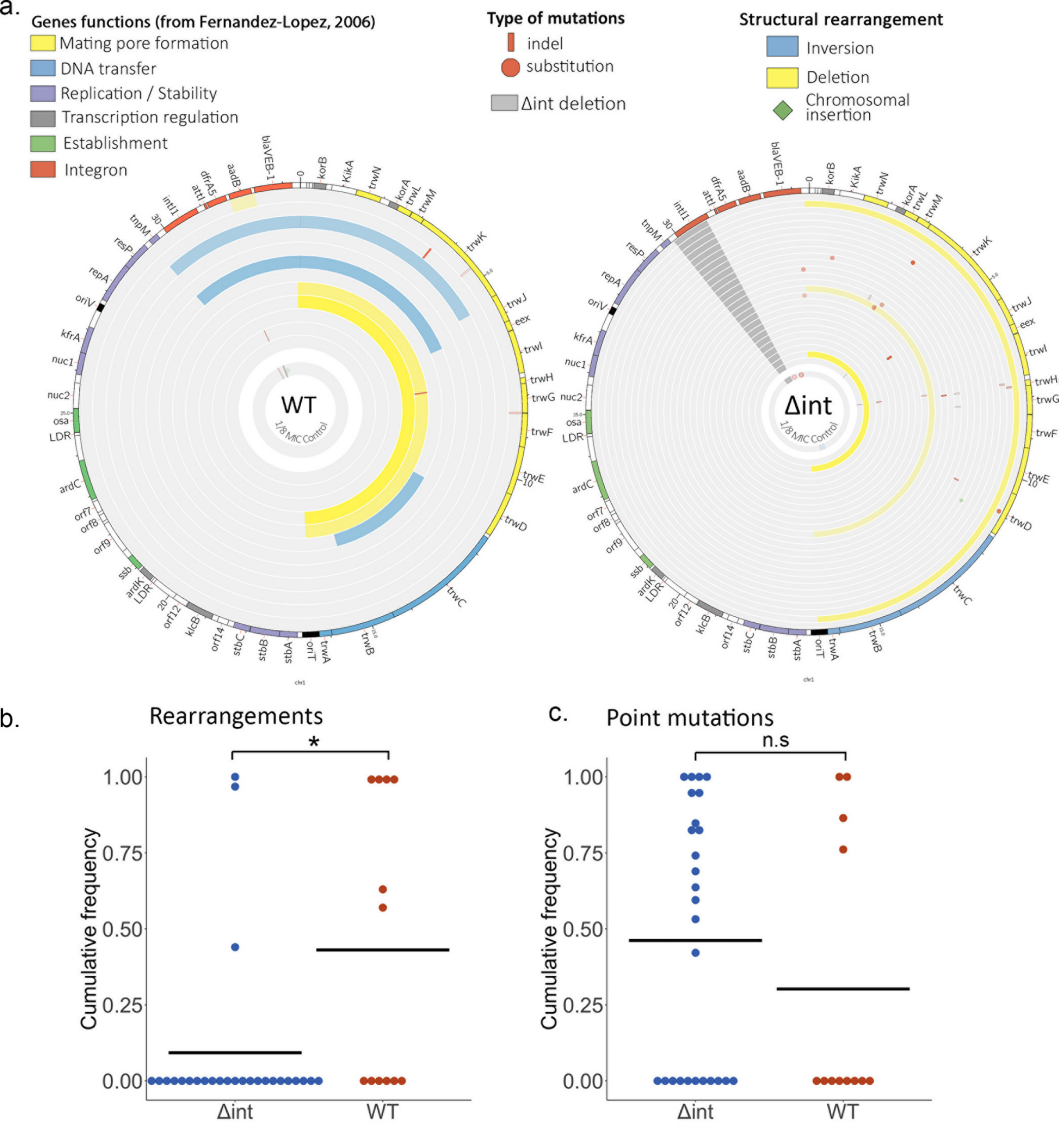
Integron activity shapes plasmid evolution through extensive structural rearrangements. (a) Representation of the plasmid mutations and rearrangements in the surviving WT populations (12 populations) (left) and Δ*int* populations (26 populations) (right) at 2× MIC, mapped to the R388 reference sequence. Each circle represents a separate population, with the inner circle representing the variants present in an equimolar pool of six 1/8× MIC control populations. Large-scale inversions and deletions are represented in blue and yellow, respectively, while indels and single nucleotide substitutions are represented in red. The color intensity represents the frequency of the corresponding mutation. The dark-gray area in the Δ*int* populations represents the location of the *intI1* deletion. As DNA was extracted from entire populations, symbols may overlap when both rearrangements and mutations are identified from separate subpopulations. The function of each R388 gene as described previously (57) is indicated by a specific color in the outer circle. (b) Cumulative frequency per population and genotype of large-scale rearrangements (inversions, deletions, and cassette rearrangements). Frequencies were compared using the Wilcoxon rank sum test (*W* = 94.5 and *P* = 0.010[*]). (c) Cumulative frequency per population and genotype of point mutations (SNPs and short indels). Frequencies were compared using the Wilcoxon rank sum test (*W* = 183 and *P* = 0.37 [n.s: non significant]).

Focusing first on the integron-bearing plasmid, we observed striking mutations and structural rearrangements targeting the conjugation machinery of the R388 plasmid backbone, with 58% (7 out of 12) of the WT and 61% (16 out of 26) of the Δ*int* populations presenting mutations and/or rearrangements in the *trw* operon (Fig. 2a). Alongside the 16.6-kb-long deletion of the DNA transfer replication and mating pore formation modules of the conjugation machinery, we also identified three large-scale inversions in this region of the plasmid: two inversions from *resP* to *trwJ* and one from *trwD* to *trwB* (9.5 kb, 10.1 kb, and 4.3 kb in size, respectively). Mutations were found in the *trwK*, *trwH*, *trwD*, *trwG*, and *trwM* genes, with 12 out of 17 mutations being either nonsense or frameshifting mutations. Finally, we identified the insertion within the *trwD* gene of one Δ*int* population of a 1.2-kbp insertion sequence (IS) element initially present on the chromosome.

In comparison, the integron array and its β-lactam cassette were much more conserved. We observed only one instance of cassette arrangement, the excision of the *aadB* cassette, in one WT population. Mutations in the integron array were also sparse, as we observed mutations and/or indels in only two Δ*int* populations (a nonsynonymous single nucleotide polymorphism [SNP] within *bla*_VEB-1_) and one WT population (a 20-bp deletion between the *attI* site and the start of the *dfrA5* cassette) (Fig. 2a and Table S1).

While the Δ*int* and WT populations evolved through modifications of similar targets, we observed a surprising difference in the way that these alterations were achieved: the conjugation machinery was disabled in Δ*int* populations mainly through mutations (Fig. 2c), while WT populations were enriched in structural rearrangements, with half of the WT populations presenting some type of rearrangement (Fig. 2b) (*W* = 94.5 and *P* = 0.010 [by a Wilcoxon rank sum test] when comparing the cumulative frequencies of rearrangements between the two genotypes). While the extensive 16-kb deletions were found in both WT and Δ*int* populations, the large-scale inversions were also specific to the populations with a functional integrase [while this could be explained by limited sampling, the chance of never observing an inversion in 24 Δ*int* populations would be around (3/4)^24 = 0.001, assuming a similar rate of inversions].

Surprisingly, we observed a different pattern, in both mechanisms and targets, in the control populations evolved at a low constant concentration of piperacillin (Fig. 2a, inner rings). The pooled controls contained predominantly mutations targeting the integron array and not the conjugation machinery, in both the WT and Δ*int* populations. We identified four nonsense and frameshifting mutations targeting the *dfrA5* cassette, two mutations in the intergenic region between the *aadB* and *bla*_VEB-1_ cassettes, one insertion within *aadB* of an IS*Pa11* element (21) initially located on the chromosome, and a 131-bp deletion within the integrase of the WT control. The only rearrangement observed was a short 773-bp inversion within *trwC* in the Δ*int* control. The comparison between variants observed in the control and piperacillin-selected populations suggests that antibiotic pressure generated selection for the loss of conjugation machinery, whereas selection in the absence of increasing antibiotic pressure drove the loss of costly resistance genes.

Finally, the plasmid was completely lost at this time point in the pooled controls passaged without antibiotic, while plasmid copy numbers remained similar between the ancestral and evolved populations of both genotypes in the ramping and constant antibiotic treatments (Fig. S2).

10.1128/mbio.02537-22.3 FIG S2 Plasmid copy number in evolved populations, estimated from sequencing data by dividing plasmid by chromosome average coverages. Control populations passaged either without antibiotics or at a constant concentration (1/8× MIC) were mixed in an equimolar pool before sequencing. Download FIG S2, TIF file, 3.6 MB. Copyright © 2023 Souque et al. 2023 Souque et al. https://creativecommons.org/licenses/by/4.0/ This content is distributed under the terms of the Creative Commons Attribution 4.0 International license.

While we observed an impact of integrase activity on plasmid evolution, no differences were identifiable on the chromosome (Table S1 and Fig. S3). WT and Δ*int* populations had average cumulative mutation frequencies on the chromosome of 0.78 and 0.66, respectively, and no extensive deletion or inversion could be identified. Parallel evolution was observed in the *PA1766–PA1768* operon, whose genes are predicted to be involved in the posttranslational modification of cell wall proteins (22), and a *PA1767* mutant was previously shown to provide a moderate increase in piperacillin resistance (23). Other mutated genes were shown to be linked with antibiotic resistance, such as *galU* (24, 25), *PA1195* (23), *clpA* (25, 26), and *infB* (25), but no link could be found for the remaining 16 of the 26 targeted genes. Excluding one mutation, all of these mutations were either nonsynonymous or intergenic and therefore still showed signs of selection, and we speculate that some of these mutations may be compensatory mutations to offset the cost of plasmid carriage. For example, we identified mutations in *hslU* and *clpA*, two ATP-binding proteases, and *rne*, an RNase, which have been shown to interact with plasmid replication proteins (27–29), a driver of plasmid cost in Pseudomonas aeruginosa (30).

10.1128/mbio.02537-22.4 FIG S3 Chromosomal mutations in the surviving WT populations (12 populations) (left) and Δ*int* populations (26 populations) (right) at 2× MIC mapped to the PAO1 reference sequence. Each circle represents a separate population. The type (indel or substitution) of each mutation is represented by the shape of the marker (lines and circles), while the marker color represents the effect of the mutation (nonsynonymous/intergenic versus synonymous), and its color intensity represents its frequency. The size of the gene labels on the outer ring represents the overall cumulative frequency of mutations present in this gene across all populations from this genotype. Download FIG S3, TIF file, 0.8 MB. Copyright © 2023 Souque et al. 2023 Souque et al. https://creativecommons.org/licenses/by/4.0/ This content is distributed under the terms of the Creative Commons Attribution 4.0 International license.

### Plasmid inversion junction sites correspond to integrase secondary sites.

**FIG 3:**
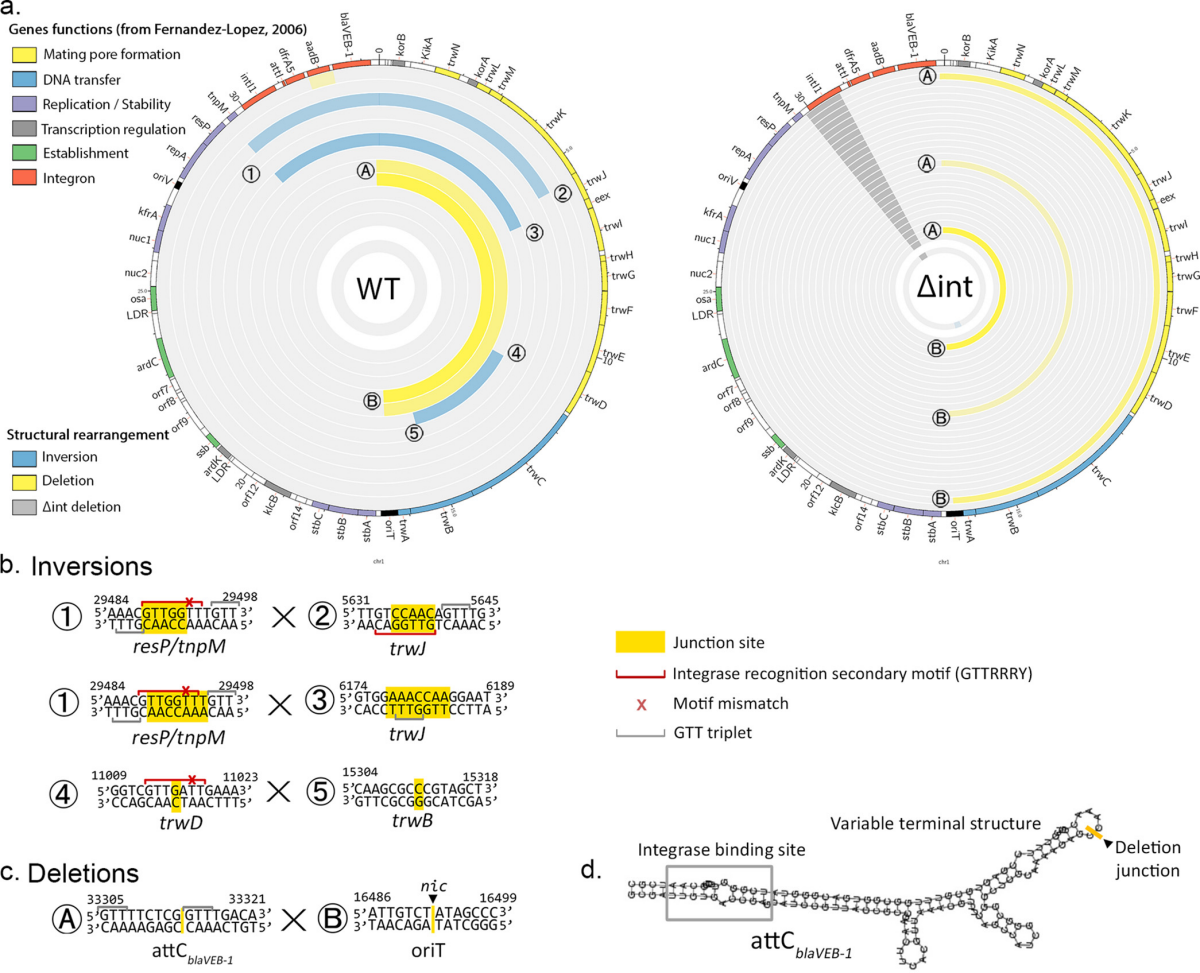
Plasmid inversion junction sites correspond to integrase secondary sites. (a) Plasmid rearrangement junctions labeled 1 to 4 (inversions) and A and B (deletions) (57). (b and c) Junction site of each inversion and deletion, based on the reference sequence. The crossover junction site is indicated in yellow. When the crossover point is unclear due to sequence homology between junctions, the entire homology is highlighted. Motifs close to the GTTRRRY integron secondary sequence are indicated by a red line, with red crosses indicating a potential mismatch. Simple 5′-GTT-3′ triplets are indicated by a gray line. The *nic* site of the origin of transfer, *oriT*, is indicated by a black triangle. (d) Folding of *attC_bla_*_VEB-1_ calculated using ViennaRNA (58), with the deletion junction in the variable terminal structure represented in yellow.

As plasmid backbone rearrangements, and especially inversions, were found more frequently in WT than in Δ*int* populations, we looked at the junction sequences for signs of integrase activity (Fig. 3). The recognition characteristics of *intI1* require little sequence identity, with only a 3-base-long conserved motif, 5′-GNT-3′, which can be extended to form the 7-bp GTTRRRY motif (31), and where cleavage usually happens between bases A and C of the complementary strand (32).

The inversions between *resP-tnpM* and *trwJ* have a strong signal of integrase activity: while the precise crossover point cannot be determined due to sequence homology, a 5′-GTT-3′ triplet, often extendable in a motif close to GTTRRRY, can be identified on both sides of the inversion. Even more strikingly, the motifs on each side of the inversion are in an inverted orientation from each other, with one located on the top strand and the other on the bottom strand. It has been shown that the integrase can create sequence inversions through intramolecular *attI* × *attI* reactions when the sites are in an inverted orientation (33) In this case, the Holliday junction formed during the first step of the recombination process is resolved through second-strand exchange instead of replication, as is the case with the canonical *attI* × *attC* or *attC* × *attC* reactions.

The involvement of the integrase in the *trwD* × *trwB* inversion is less straightforward. We identified a motif close to GTTRRRY at the *trwD* end but with a cleavage point after the 5′-GTT-3′ triplet. It has been shown that the constraints on the second nucleotide of the triplet are fairly lax (8, 32) and that cleavage can happen between the G and the A of a 5′-GAT-3′ triplet, as observed here, especially in the case of reactions with secondary sites (34). However, we were not able to identify any 5′-GTT-3′ triplet in the vicinity of the *trwB* junction. As a control, we also investigated the small inversion located within the 1/8× MIC Δ*int* pooled sample: we identified one 5′-GTT-3′ triplet on one side but not on the other and no motif close to GTTRRRY.

In contrast, extended deletions found in both WT and Δ*int* populations have the same boundary site, which does not show integrase-binding motifs. One boundary is found in *attC_bla_*_VEB-1_ and its variable terminal structure, a part of the *attC* site usually not involved in integrase recognition. The other end of the deletion is located precisely at the *nic* site of the R388 *oriT*, where the *trwC* relaxase binds during conjugation and initiates the nicking of the DNA to create single-stranded DNA, which is then exported to the recipient cell and recircularized. It has been shown that *trwC* can mediate site-specific recombination between two *oriT* sites even in the absence of conjugation (35), which leads to the loss of the intervening DNA (36). It is unclear if the *trwC* enzyme can bind to the sequence at the *attC_bla_*_VEB-1_ junction site: the recognition of the *nic* site is supposed to be highly specific (37), and no sequence homology could be seen between *oriT* and *attC_bla_*_VEB-1_. As the limiting step for *oriT* recombination is the generation of a single strand at *oriT* to allow *trwC* nicking (38), recombination between the two sites may be facilitated by the *attC* site’s unique secondary structure, which makes the junction site available as single-stranded DNA.

### Integron activity alleviates plasmid fitness costs and improves plasmid persistence through loss of the conjugation machinery.

**FIG 4:**
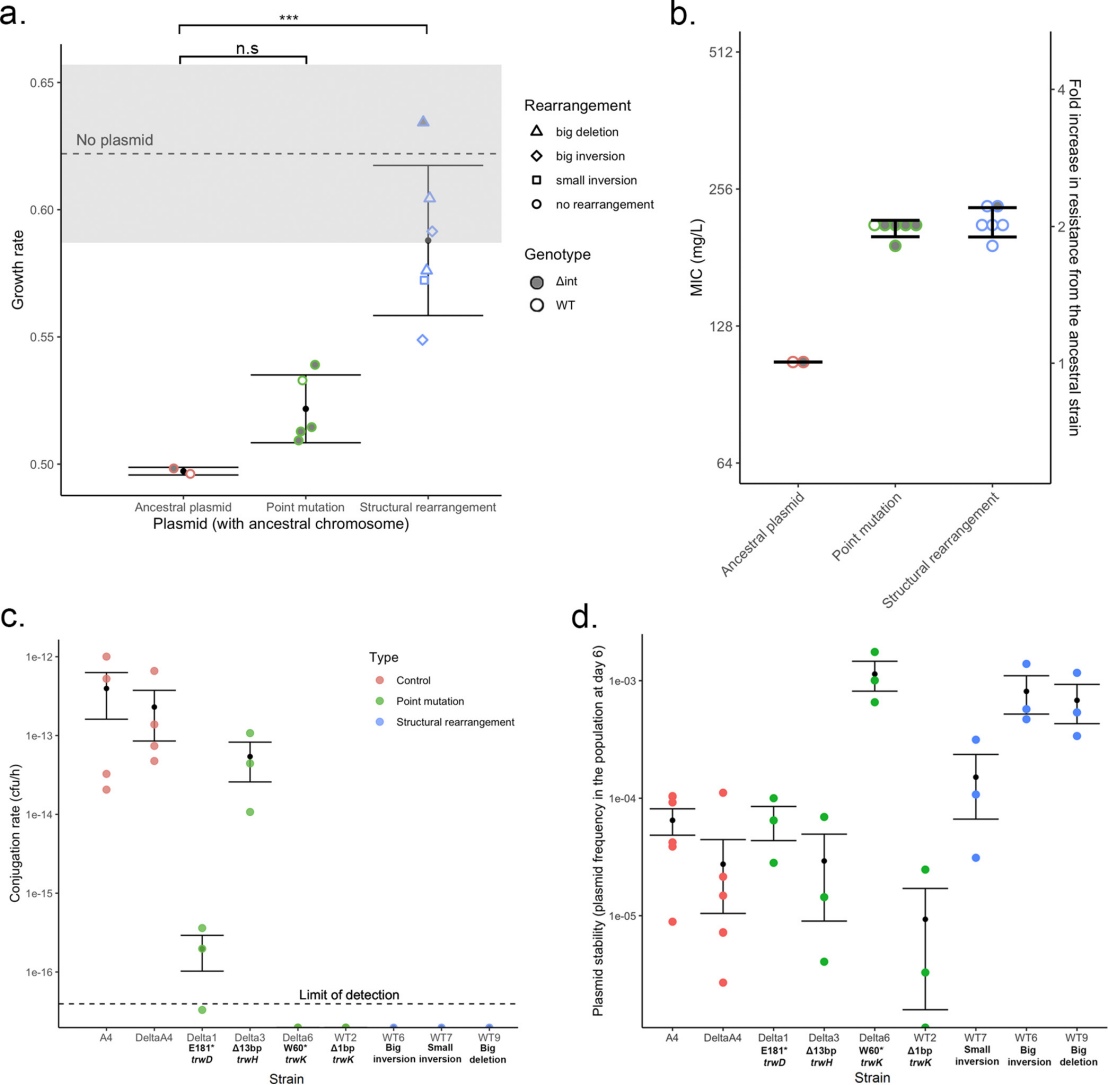
Loss of the conjugation machinery increases piperacillin resistance and improves plasmid persistence. (a and b) Growth rate in the absence of antibiotics (a) and piperacillin MICs (b) of the evolved plasmids when transferred back into the ancestral chromosome. Each dot represents the average from three independent biological replicates. The error bars correspond to the standard deviations with each category. The gray area represents the standard error of the growth rate in the absence of the plasmid. Growth rates between genotypes were compared using linear regression (*F* = 17.929 and *P* < 0.0005), and coefficients were compared to those of the ancestral plasmid control (*t* = 1.301 and *P* > 0.2 for point mutations [n.s: non significant], and *t* = 4.935 and *P* < 0.001 [***] for structural rearrangement populations [by a *t* test]). (c) Conjugation rate estimation for a selection of mutations and rearrangements after filter mating. The dotted line corresponds to the average sensitivity limit of the assay. Results from one (structural rearrangements) to three (point mutations) biological replicates are shown. (d) Plasmid stability estimated by the final plasmid frequency obtained after transferring 3 to 5 independent populations of each strain in the absence of antibiotics for 6 days. Error bars represent standard errors.

A key insight from the sequencing of the evolved populations is that integrase-containing populations tend to evolve by structural rearrangements of the R388 conjugative apparatus, whereas Δ*int* populations evolve via point mutations in conjugative genes. We then investigated if the higher proportion of structural rearrangements in populations with a functional integrase could explain the higher final fitness observed previously. To test this idea, we extracted the plasmids from subsets of WT and Δ*int* evolved populations and transformed them into the ancestral chromosomal background. We found that the ancestral R388 plasmid imposes a high fitness cost, and this cost was ameliorated by compensatory evolution during the piperacillin selection experiment (Fig. 4a). Crucially, plasmids with extensive rearrangements of the conjugation machinery imposed a lower fitness cost than the unevolved plasmids or plasmids with point mutations. In contrast, both mutations and rearrangements provided similar levels of resistance to piperacillin (Fig. 4b).

The strong compensatory effects associated with structural rearrangements suggest that these variants increased fitness by eliminating the conjugative ability of the R388 plasmid. To test this hypothesis, we measured the conjugation rate of evolved plasmids. Both inversions and deletions were enough to stop the plasmid from conjugating, while mutations often provided an intermediate phenotype (Fig. 4c).

Both selection and horizontal transfer impact the ability of plasmids to persist in bacterial populations. The observed structural rearrangements decrease the fitness costs of plasmid carriage, which should improve the ability of the plasmid to persist through vertical transmission. However, these rearrangements also reduce the conjugative ability, hindering the ability of R388 to persist via horizontal transfer. To understand the net effect of plasmid evolution on stability, we transferred independent populations of each plasmid in the absence of antibiotics for 6 days and measured the ratio of cells containing the plasmid at the final time point. Rearranged plasmids were present at a 10-fold-higher frequency than ancestral or mutated plasmids, showing that these rearrangements improved plasmid persistence (Fig. 4d).

## DISCUSSION

The adaptation-on-demand model shows the clear benefits of the integron when cassettes have high mobility and the cassette position is under strong selection. However, it is important to emphasize that integron cassettes display a diversity of fitness costs, recombination rates, and positional effects, all of which may impact the benefits of integrase activity. Using the β-lactamase-encoding cassette *bla*_VEB-1_, which has been shown to have reduced mobility and a weak cassette expression gradient, we investigated the evolutionary impact of integrase activity in an experiment where selection for cassette rearrangement is weak. Under these conditions, integrase expression decreased the survival rates of the populations under antibiotic treatment, revealing a cost to integrase activity (Fig. 1c). However, the integrase had a positive impact on the fitness of the surviving populations by generating inversions in conjugative genes that effectively compensated for the high costs associated with the R388 plasmid (Fig. 2a). Integrase-mediated compensatory adaptation increased the stability of the R388 plasmid in the absence of antibiotic pressure albeit with the cost of a decreased conjugative ability (Fig. 4d). These results highlight the limitation of the adaptation-on-demand model, and they emphasize the potential importance of off-target effects to moderate the fitness costs and benefits of the integron.

Our study showed that the potential benefits of integrase activity are not limited to the canonical *attI* × *attC* and *attC* × *attI* reactions but also can lead to adaptive inversions between secondary sites through *attI* × *attI*-like reactions. The mechanism behind the reactions between *attI* sites was studied in detail previously (33), but its potential adaptive benefits have remained elusive. *attI*-like sites can be found frequently across genomes (16), and these adaptive rearrangements between secondary sites may be common. Integrons consisting of only an integrase and without cassette arrays (so-called *In0*) have been found several times across bacterial genomes (39, 40), and the adaptive benefits of off-target integrase activity may help to maintain these cassetteless integrons.

While the inversions that we observed had clear signs of integrase activity, the mechanism behind the extensive *attC* × *oriT* deletions that we observed is more elusive. The relaxase *trwC* has been shown to mediate site-specific excision between two *oriT* sites and is triggered by the generation of single-stranded DNA at one of the *oriT* sites (38). The unique single-stranded folding of *attC* sites may make them unexpected hot spots for recombination through their variable terminal structures (VTSs). The ubiquity of integron cassettes on plasmids suggests that integron cassettes and their *attC* sites may play a role in plasmid remodeling, even in bacteria containing only dysfunctional integron integrase pseudogenes (41).

When comparing these results to those of our previous study, it is noteworthy that with the *aadB* and *bla*_VEB-1_ cassettes, the intensity of the cassette expression gradient depending on the position in the array was positively correlated with mobility. Depending on the resistance mechanism that they encode, not all cassettes may be able to generate a significant resistance gradient in the typically 1- to 6-cassette length of a mobile integron (for example, *dfrA5*). Similarly, higher expression levels that could be provided by cassette duplication may be deleterious, as is the case for some β-lactamase cassettes (42). In that case, and especially if the cassette has a high fitness cost, a highly mobile *attC* site would have little benefit to the cassette but would be linked to a higher rate of cassette excision, leading to the selection of *attC* sites with lower excision rates. Integron cassettes as mobile genetic elements may be subject to their own level of selection, leading to traits that may not be constantly beneficial for their bacterial host or the integrase itself.

Finally, this study showed the striking parallel evolution of conjugation-deficient plasmids in response to antibiotic selection pressure. R388 was found to be a very costly plasmid for P. aeruginosa, but its fitness cost could be greatly improved by disabling the conjugation machinery. It is not surprising that conjugative genes carry a fitness cost as they are often associated with an increased cellular burden (43, 44). In P. aeruginosa, it has been shown that the conjugative pilus causes a membrane perturbation that triggers the “tit-for-tat” response and activates the type VI secretion system (T6SS) (45), while conjugative genes can also be targeted by CRISPR-Cas systems (46). Not all conjugative plasmids are costly to P. aeruginosa, with plasmid pAKD1 containing tightly repressed conjugation genes that actually increase PAO1 fitness (47), but a poorly regulated conjugation machinery could generate a cascade of costly reactions. Surprisingly, we found that disrupting the conjugative machinery led to an unexpected increase in piperacillin resistance, revealing a novel cost of conjugation independently of its impact on P. aeruginosa fitness. We can speculate that the presence of conjugative pili on the bacterial membrane may make the bacteria more susceptible to β-lactam antibiotics. This effect is relatively subtle (2× change in MIC), but it clearly led to the accelerated loss of conjugative genes in the presence of antibiotics. The generality of this mechanism is unclear, but our results raise the tantalizing possibility that antibiotic conjugation-induced susceptibility may be an unexplored selection pressure driving the acquisition and maintenance of β-lactam resistance genes on conjugative plasmids.

## MATERIALS AND METHODS

### Strains and growth conditions.

The strains and plasmids used in this paper are listed in Table S2 in the supplemental material. Unless stated otherwise, bacteria were grown in LB Miller broth at 37°C with shaking (225 rpm), and plasmid maintenance was guaranteed by the addition of 100 mg/L of ceftazidime.

10.1128/mbio.02537-22.6 TABLE S2 List of strains and plasmids used in this study. Download Table S2, XLSX file, 0.01 MB. Copyright © 2023 Souque et al. 2023 Souque et al. https://creativecommons.org/licenses/by/4.0/ This content is distributed under the terms of the Creative Commons Attribution 4.0 International license.

### MIC determination.

The MICs of each antibiotic were determined using the broth microdilution method according to Clinical and Laboratory Standards Institute (CLSI [59]) guidelines. Inocula containing 5 × 10^5^ CFU of bacteria were prepared in cation-adjusted Mueller-Hinton broth 2 (MH2) using individual colonies grown on selective agar and incubated with doubling concentrations of antibiotics for 20 h in three technical replicates. The culture optical density at 600 nm (OD_600_) was then read using a BioTek Synergy plate reader, and wells were considered empty when the overall OD_600_ was below 0.1. The MIC for each assay was defined as the minimal concentration at which growth was inhibited in all three technical replicates. The final MIC values are the averages from two to three replicate assays (from separately prepared inocula, on different days).

### Growth rate determination.

Growth rates in the absence of antibiotics were determined using a BioTek Synergy plate reader. Populations were inoculated under conditions similar to those for the MIC assay (inocula of 5 × 10^5^ CFU of bacteria prepared in cation-adjusted MH2 from individual colonies) and monitored for 24 h at 37°C with periodic shaking, with OD_600_ measurements being taken every 15 min. Intrinsic growth rates were determined using the Growthcurver package (48). Each measurement was done in three technical replicates (separate wells from the same inoculum) and replicated three times over different days (biological replicates).

### Experimental evolution.

Ninety populations of each strain (PAO1::WTA4 and PAO1::Δ*intA4*) were initially inoculated from individual colonies grown on selective agar in MH2 at a piperacillin concentration of 1/8× MIC. The outer wells of every plate were kept as negative controls, and the strain distribution among the plates was kept balanced to limit plate effects. Every day, the populations were diluted 1/10,000 with a doubling concentration of the MIC until 8× MIC was reached. As controls, 15 populations of each strain were transferred either without antibiotics or at a constant piperacillin concentration of 1/8× MIC. The OD of each population was measured each day, and a population was considered extinct when its OD fell below 0.1 after 20 h of incubation. All populations were frozen in 15% glycerol every 2 days.

### Genome analysis.

**(i) DNA extraction.** PAO1::WTA4 and PAO1::Δ*int*A4 populations from the 2× MIC time point were regrown from frozen stocks in LB Miller medium supplemented with a piperacillin concentration of 1× MIC (ramping populations) or 1/8× MIC (1/8× MIC controls), no antibiotic (no-antibiotic controls), or 100 mg/L of ceftazidime (ancestral PAO1::WTA4 and PAO1::Δ*int*A4 populations). DNA extraction was performed using the DNeasy blood and tissue kit (Qiagen). For each control treatment (1/8× MIC and no antibiotic, for each genotype), the DNAs from six populations were pooled in equimolar ratios and sequenced as one sample.

**(ii) Next-generation sequencing.** Library preparation and sequencing were performed by the Oxford Genomics Centre at the Wellcome Centre for Human Genetics in two batches. Twelve samples of each genotype, the ancestral populations (PAO1, PAO1::WTA4, and PAO1::Δ*int*A4), and the pooled controls were first sequenced on the NextSeq 500 sequencing system (Illumina). Five PAO1::WTA4 populations as well as the ancestral strains were contaminated by phages during the DNA extraction process and were reextracted and resequenced alongside the remaining PAO1::Δ*int*A4 populations (14 populations) on the HiSeq4000 platform.

**(iii) Bioinformatic pipeline.** PCR duplicates and optical artifacts were removed using MarkDuplicates (Picard toolkit [49]), while low-quality bases and adaptors were trimmed using Trimmomatic v.039 (50), and overall quality control was performed using FastQC (51) and multiQC (52). Phage contamination in the first batch of samples was determined at this stage.

Variant calling and rearrangement identifications were performed using the breseq pipeline in polymorphism mode (53). Variants present in the unevolved ancestor populations were filtered out irrespective of frequency. Due to the lower quality of the samples processed by the NextSeq 500 system, which led to an overidentification of low-frequency variants, a filtering process harsher than the one in our previous analysis (5) was carried out: any variant present in the controls evolved without antibiotics or at a frequency of <30% was removed from the ramping population data set. The evidence for any variant above this threshold and present in several NextSeq 500 populations was manually examined, and the variant was removed if present in a region of low read quality. For the pooled 1/8× MIC populations, a lower threshold of 5% (which corresponds to a variant present in 30% of 1 out of 6 populations) was applied, and manual examination was carried out to exclude low-quality variants. Additional evidence for plasmid rearrangements identified by breseq (large-scale deletions, inversions, or chromosomal insertions) was obtained using plasmidSPAdes (54). Finally these rearrangements were confirmed by PCR (Text S1, Table S3).

### Analysis of evolved plasmids.

**(i) Conjugation rate determination.** Determination of the conjugation rates of the ancestral and evolved plasmids was performed using filter mating with a rifampicin-resistant PAO1 acceptor (D512G *rpoB* mutant reported previously [55]). Populations of donors and recipients were grown overnight for 3 h 30 min with (donors) and without (recipient) antibiotics, washed, and resuspended in NaCl (0.9%). Donor and recipient populations were mixed at a 1:1 ratio, deposited onto a filter on an agar plate without antibiotics alongside the pure donor/recipient control, and incubated for 1 h. Populations were then resuspended in 0.9% NaCl. Concentrations of donors, recipients, and transconjugants were determined at both initial (*T*_0_) and final (*T_f_*) timepoint using the appropriate antibiotics (ceftazidime at 100 mg/L, rifampicin at 64 mg/L, and both combined). The conjugation rate was calculated using the method of Simonsen et al. (56).

**(ii) Plasmid maintenance over time.** Populations with ancestral and evolved plasmids were inoculated from single colonies in the presence of antibiotics and passaged for 6 days in the absence of antibiotics at a dilution (1:10,000) similar to the on used for the experimental evolution experiment. After 6 days, the ratio of plasmid-containing cells was determined by serial dilution plating with (for the CFU of the plasmid-containing cells) and without (for the CFU of the entire population) ceftazidime. The presence of horizontal gene transfer under these conditions was confirmed by mixing a subset of the populations with rifampicin-resistant bacteria after day 1 and plating them after 20 h on rifampicin- and ceftazidime-containing plates.

### Data availability.

Sequencing reads have been deposited at the ENA under accession number PRJEB52820. Source data files have been deposited at Dryad (https://doi.org/10.5061/dryad.fj6q573xq). Strains and plasmids generated in this study are available upon request.

10.1128/mbio.02537-22.1 TEXT S1 MIC determination under experimental conditions, PCR analysis, and transformation of the evolved plasmids into the ancestral chromosomal background. Download Text S1, DOCX file, 0.01 MB. Copyright © 2023 Souque et al. 2023 Souque et al. https://creativecommons.org/licenses/by/4.0/ This content is distributed under the terms of the Creative Commons Attribution 4.0 International license.

10.1128/mbio.02537-22.7 TABLE S3 List of primers used in this study. Download Table S3, XLSX file, 0.01 MB. Copyright © 2023 Souque et al. 2023 Souque et al. https://creativecommons.org/licenses/by/4.0/ This content is distributed under the terms of the Creative Commons Attribution 4.0 International license.
