# Supplementary figures and images for: Off-Target Integron Activity Leads to Rapid Plasmid Compensatory Evolution in Response to Antibiotic Selection Pressure
Source: mBio. 2023 Feb 22;14(2):e02537-22. doi: 10.1128/mbio.02537-22 (PMC10127599; doi:10.1128/mbio.02537-22)

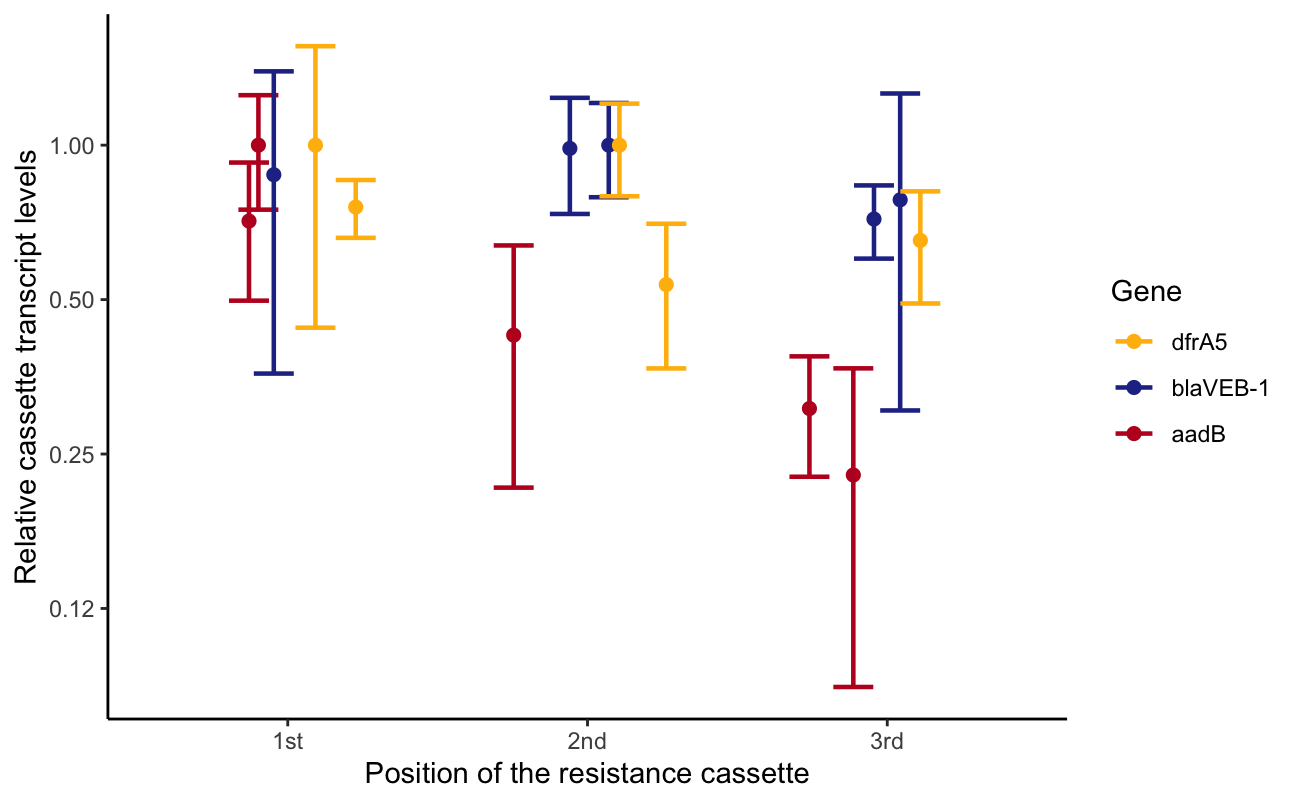

Supplement: FIG S1 [file mbio.02537-22-s0001.tif]

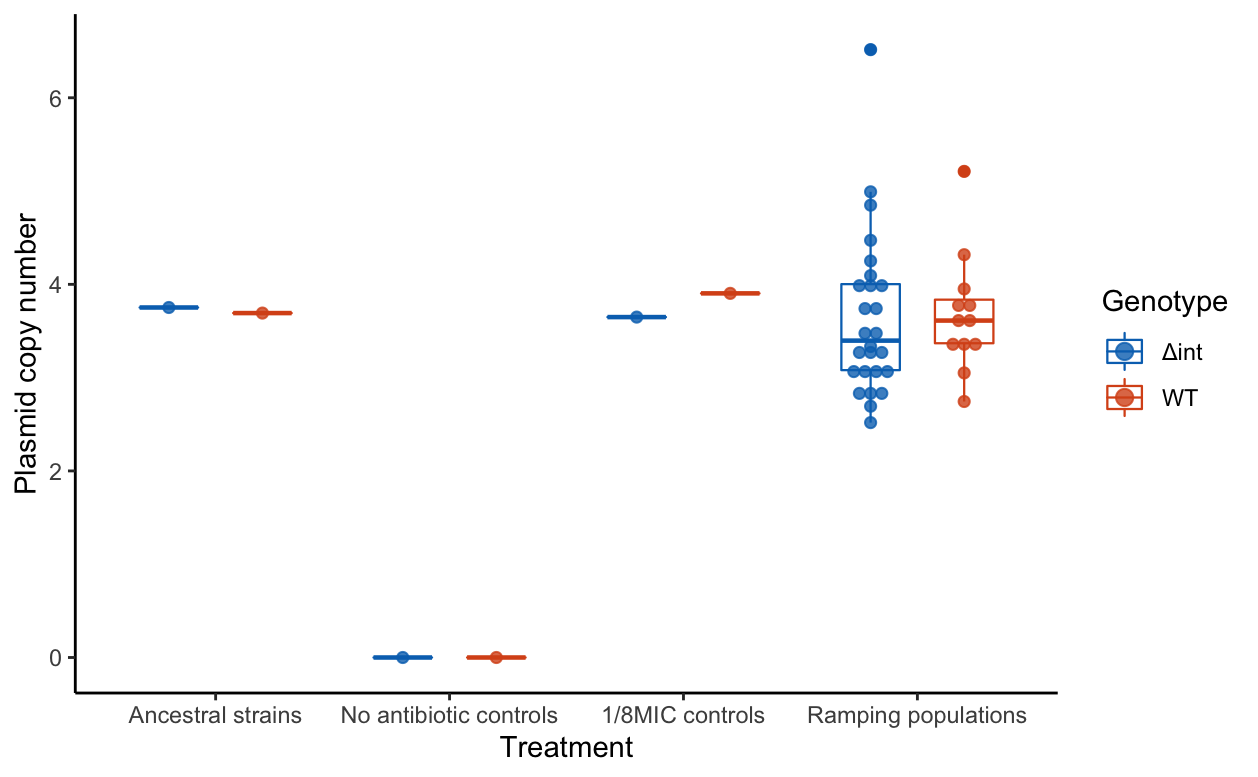

Supplement: FIG S2 [file mbio.02537-22-s0002.tif]

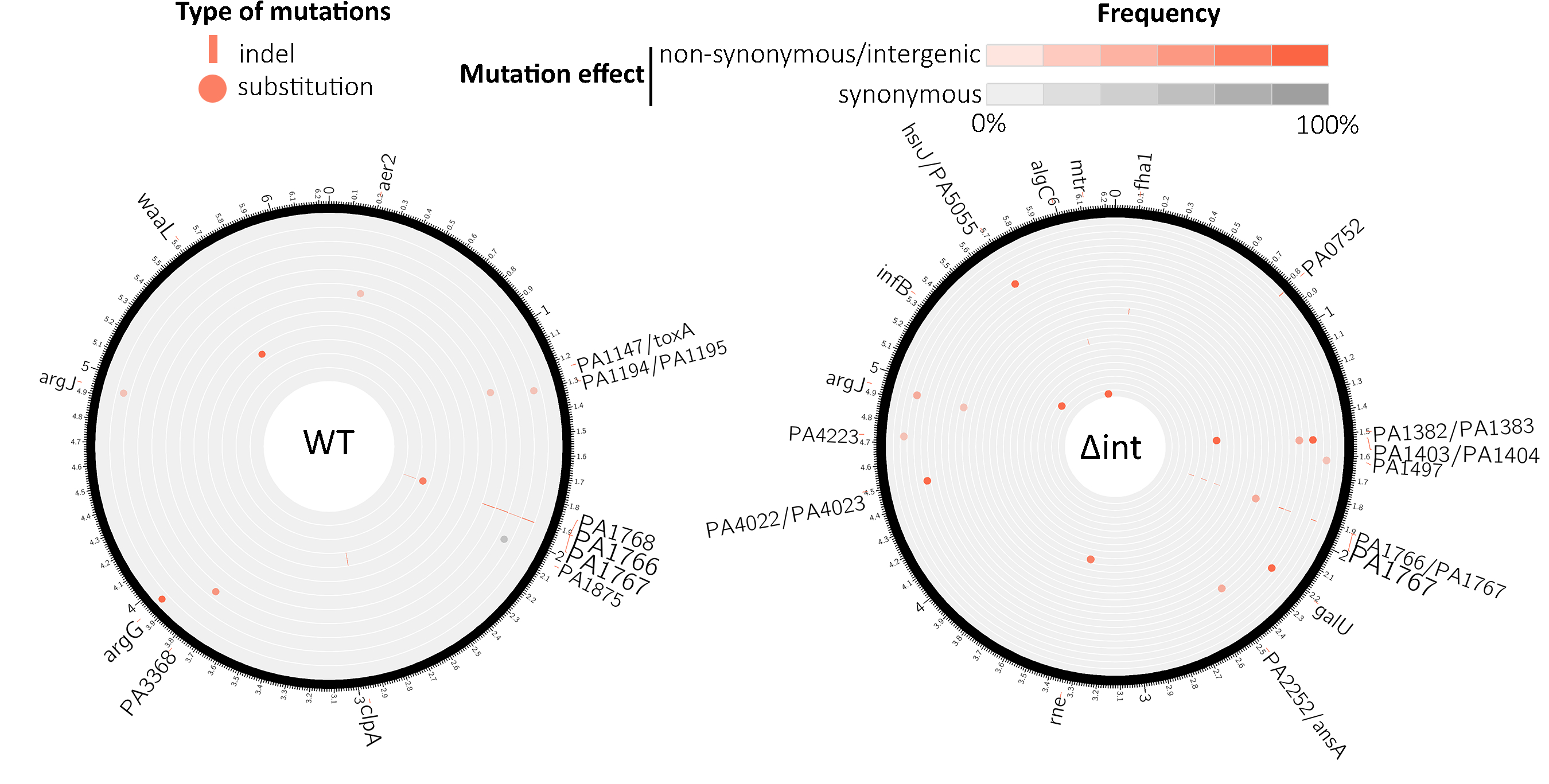

Supplement: FIG S3 [file mbio.02537-22-s0003.tif]
