# Supplementary material for: Off-Target Integron Activity Leads to Rapid Plasmid Compensatory Evolution in Response to Antibiotic Selection Pressure
Source: mBio. 2023 Feb 22;14(2):e02537-22. doi: 10.1128/mbio.02537-22 (PMC10127599; doi:10.1128/mbio.02537-22)
Supplement: TEXT S1 [file mbio.02537-22-s0007.docx]

**Supplementary Methods**

Minimum inhibitory concentration in experimental conditions

Minimum inhibitory concentrations for piperacillin were determined for all strains in conditions matching the experimental evolution set-up: overnight cultures were diluted 1/10 000 and supplemented with doubling concentrations of piperacillin. MICs were determined after 20h of incubation. This process was repeated twice. In these conditions the MIC were 64 mg/L of piperacillin for both the PA01:WTA4 and PA01:*Δint*A4 strains.

PCR analysis

Cassette rearrangement and structural variants were also confirmed by PCR. Surviving populations at the x2MIC time point were plated using a pin replicator on LB agar supplemented with piperacillin at a concentration corresponding to x1 MIC and incubated for 48h at 37°C. 12 populations of each genotype were picked randomly, boiled for 10 minutes at 95°C in distilled water and screened for cassette rearrangement. The cassette rearrangement panel consisted of 4 reactions: amplification of the integrase size (as control for cross-contamination), localisation of the *blaVEB-1* cassette relative to the start of the integrase, localisation of the *blaVEB-1* cassette relative to the end of the *dfrA5* cassette and identification of potential *blaVEB-1* cassettes duplications (primer sequences are indicated in Table S3). These PCRs were performed using the GoTaq G2 DNA mastermix (Promega) for 30 cycles with 30s at 95°C denaturation, 30s at 55°C annealing and 3 minutes at 72°C elongation.

The inversions and deletions identified through whole genome sequencing (inversions between *resP* x *trwJ* and *trwD x trwB*, deletions between *blaVEB-1* and *stbA)* were confirmed through a second PCR panel. Inversions were detected using a pair of primers specific to the integrase and *trwG* gene, or the t*rwD* and *trwB* genes, and binding on the same strand, therefore only producing an amplicon if the intervening sequence is reversed. Deletions were targeted using primers specific to *blaVEB-1* and *stbA*: given *blaVEB-1* and *stbA* are separated by 17 kbp on the ancestral plasmid, amplification is only possible in the presence of deletion bringing the primers closer together. The following PCR protocol was used: 30s at 95°C denaturation, 30s at 55°C annealing and 1 minute at 72°C elongation.

**Transformation of the evolved plasmids into the ancestral chromosomal background**

Extraction of the plasmids from the evolved populations was performed using the QIAprep Miniprep (Qiagen) on the QiaCube extraction platform from liquid culture from the frozen populations grown in LB Miller supplemented with 32mg/L of piperacillin. Extracted plasmids were then electroporated back into PA01 (Choi and Schweizer, 2006). Presence of the plasmid was confirmed by PCR. Colony PCR and, when required, Sanger sequencing of the amplicons was performed to identify single colonies containing either the rearrangements or the selected mutations.

**References**

Choi, KH., Schweizer, H. mini-Tn7 insertion in bacteria with single attTn7 sites: example Pseudomonas aeruginosa. Nat Protoc 1, 153–161 (2006). https://doi.org/10.1038/nprot.2006.24
